# Aflatoxin B1 and Sterigmatocystin Binding Potential of Non-*Lactobacillus* LAB Strains

**DOI:** 10.3390/toxins12120799

**Published:** 2020-12-14

**Authors:** Ildikó Bata-Vidács, Judit Kosztik, Mária Mörtl, András Székács, József Kukolya

**Affiliations:** 1 Department of Environmental and Applied Microbiology, Agro-Environmental Research Institute, National Agricultural Research and Innovation Centre, 1022 Budapest, Hungary; kosztik.judit@akk.naik.hu (J.K.); kukolya.jozsef@gmail.com (J.K.); 2 Department of Environmental Analysis, Agro-Environmental Research Institute, National Agricultural Research and Innovation Centre, 1022 Budapest, Hungary; mortl.maria@akk.naik.hu (M.M.); szekacs.andras@akk.naik.hu (A.S.)

**Keywords:** aflatoxin B1, sterigmatocystin, lactic acid bacteria, mycotoxin binding, detoxification

## Abstract

Research on the ability of lactic acid bacteria (LAB) to bind aflatoxin B1 (AFB1) has mostly been focusing on lactobacilli and bifidobacteria. In this study, the AFB1 binding capacities of 20 *Enterococcus* strains belonging to *E. casseliflavus*, *E. faecalis*, *E. faecium*, *E. hirae*, *E. lactis*, and *E. mundtii*, 24 *Pediococcus* strains belonging to species *P. acidilactici*, *P. lolii*, *P. pentosaceus*, and *P. stilesii*, one strain of *Lactococcus formosensis* and *L.* *garviae*, and 3 strains of *Weissella soli* were investigated in MRS broth at 37 °C at 0.2 µg/mL mycotoxin concentration. According to our results, among non-lactobacilli LAB, the genera with the best AFB1 binding abilities were genus *Pediococcus*, with a maximum binding percentage of 7.6% by *P. acidilactici* OR83, followed by genus *Lactococcus*. For AFB1 bio-detoxification purposes, beside lactobacilli, pediococci can also be chosen, but it is important to select a strain with better binding properties than the average value of its genus. Five *Pediococcus* strains have been selected to compare their sterigmatocystin (ST) binding abilities to AFB1 binding, and a 2–3-fold difference was obtained similar to previous findings for lactobacilli. The best strain was *P. acidilactici* OR83 with 18% ST binding capacity. This is the first report on ST binding capabilities of non-*Lactobacillus* LAB strains.

## 1. Introduction

Mycotoxins are secondary metabolites produced by microfungi that are capable of causing disease and death in humans and other animals [1]. The effects of some food-borne mycotoxins are acute with symptoms of severe illness appearing rapidly after consumption of food products contaminated with mycotoxins [2]. Of the several hundred mycotoxins identified so far, about a dozen have gained the most attention due to their severe effects on human health and their occurrences in food; and among the most commonly observed mycotoxins that present a concern to human health and livestock are aflatoxins [2].

Aflatoxins are amongst the most poisonous mycotoxins produced by species within *Aspergillus* section *Flavi*, which grow in soil, decaying vegetation, hay, grains, and various other substances [3,4]. Crops that are frequently affected by *Aspergillus* spp. include cereals (corn, sorghum, wheat, and rice), oilseeds (soybean, peanut, sunflower, and cotton seeds), spices (chili peppers, black pepper, coriander, turmeric, and ginger), and tree nuts (pistachio, almond, walnut, coconut, and Brazil nut) [1,5,6,7,8,9]. The four major aflatoxins are called aflatoxins B1, B2, G1, and G2 based on their fluorescence under UV light (blue or green). Among them, aflatoxin B1 (AFB1) is one of the most hazardous mycotoxins, primarily carcinogenic and genotoxic [10] and harmful to the liver [11]. The International Agency for Research on Cancer (IARC) classifies AFB1 as Group 1 carcinogen (carcinogenic to humans) [12]. In accordance with Regulation (EU) No 574/2011, the maximum permitted level for AFB1 in feed is 0.02 mg/kg.

Sterigmatocystin (ST) is a late metabolite in the aflatoxin pathway and is also produced as a final biosynthetic product by a number of species such as *Aspergillus versicolor* and *Aspergillus nidulans* [1,13]. ST is both mutagenic and tumorigenic but is less potent than aflatoxin [13]. Although experiments have shown genotoxicity and carcinogenicity of ST, limited data are available on the tumorigenic effect of the mycotoxin, which is why IARC classified it as a potential human carcinogen (Group 2B) in 1987 and has not revised this opinion ever since [14].

Lactic acid bacteria (LAB), Gram-positive, nonsporulating, oxidase and catalase negative, anaerobic aerotolerant microorganisms, are found in both the animal and the human body [15]. They ferment glucose to lactic acid. The most important genera are *Lactobacillus*, *Lactococcus*, *Leuconostoc*, *Enterococcus*, and *Pediococcus*, with 294, 21, 15, 59, and 11 species belonging to each genus, respectively [16].

Food-borne lactic acid bacteria able to bind mycotoxins can prevent their biotransformation to more toxic metabolites in the digestive tract, as the mycotoxin-microorganism adduct can pass through the body and be excreted in the feces, similarly as in the case of industrial mycotoxin binders, like aluminosilicates or glucomannan [17,18]. Numerous studies have shown that certain strains of some LAB species can bind mycotoxins, among them AFB1, to their surface [19,20,21]. The published results indicate that the adsorption of AFB1 to microorganisms is a rapid process. The binding involves the formation of a reversible complex between the chemically unmodified mycotoxin molecule and the microorganism surface, and the yield of AFB1 removal is dependent of the concentration of both the mycotoxin and the bacteria [22]. The binding mechanism is not yet elucidated, but the binding of AFB1 to the glycan components of the cell wall of probiotic bacteria has been suggested as a key momentum in the process [23,24,25].

According to literature data, the binding of aflatoxins by LAB strains is highly strain specific. The ratio of AFB1 bound by 10^9^ cfu/mL of 8 strains of *L. casei* varied from 14% to 49% from the available 4.6 µg/mL in the studies of Hernandez-Mendoza et al. [26]. Reasons for the strain-specificity of AFB1 binding are yet unknown, but differences in cell wall components, particularly in the peptidoglycan content, may be implicated [27].

There has been quite a bit of research done on the ability of LAB to mitigate the detrimental effects of aflatoxin-producing fungal strains and their AFB1 binding capacity [20,22,28,29,30], though focusing mostly on lactobacilli and bifidobacteria. In addition, data on aflatoxin binding abilities of different strains belonging to the same species are scarce.

At our department, microbes with colony morphology of lactic acid bacteria were isolated on LAB selective MRS (de Man, Rogosa and Sharpe, VWR) plates from 14 exotic animals of the Budapest Zoo and Botanical Garden [31,32]. The identification of the strains was done by sequencing. At present, the collection comprises of nearly 1000 strains and is constantly expanding. Most of our strains belong to the genera *Lactobacillus* and *Enterococcus*, but we also managed to isolate strains belonging to the other LAB genera.

Our goal was to screen strains of the genus *Enterococcus*, *Lactococcus*, *Pediococcus*, and *Weissella* from our collection for AFB1 and ST binding capacities. In the literature, PBS medium (phosphate buffer solution) is most commonly used in mycotoxin binding assays of LAB strains. As our work was carried out as part of a probiotic development project, we chose a medium, MRS medium (Lactobacillus Agar according to DeMan, Rogosa and Sharpe), for our experiments, which is closer to real conditions due to its much higher organic matter content and provides optimal conditions for the microbes. Mycotoxin concentrations were monitored in rapid analysis by an instrumental method, high-performance liquid chromatography (HPLC) coupled with UV detection of the target analytes, AFB1 and ST, upon solvent extraction. Separation of the mycotoxins was achieved on hydrophobic linear alkylsilane stationary phase.

## 2. Results and Discussion

### 2.1. Instrumental Analysis of Mycotoxins

**Figure 1:**
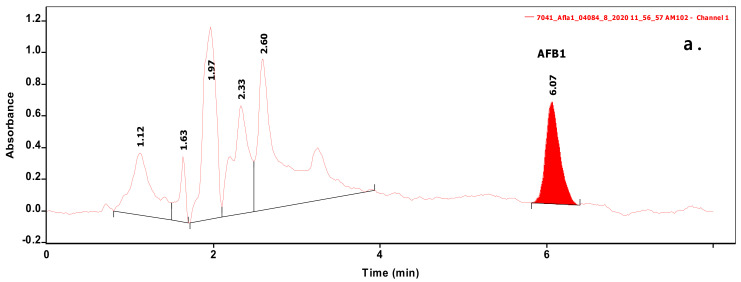

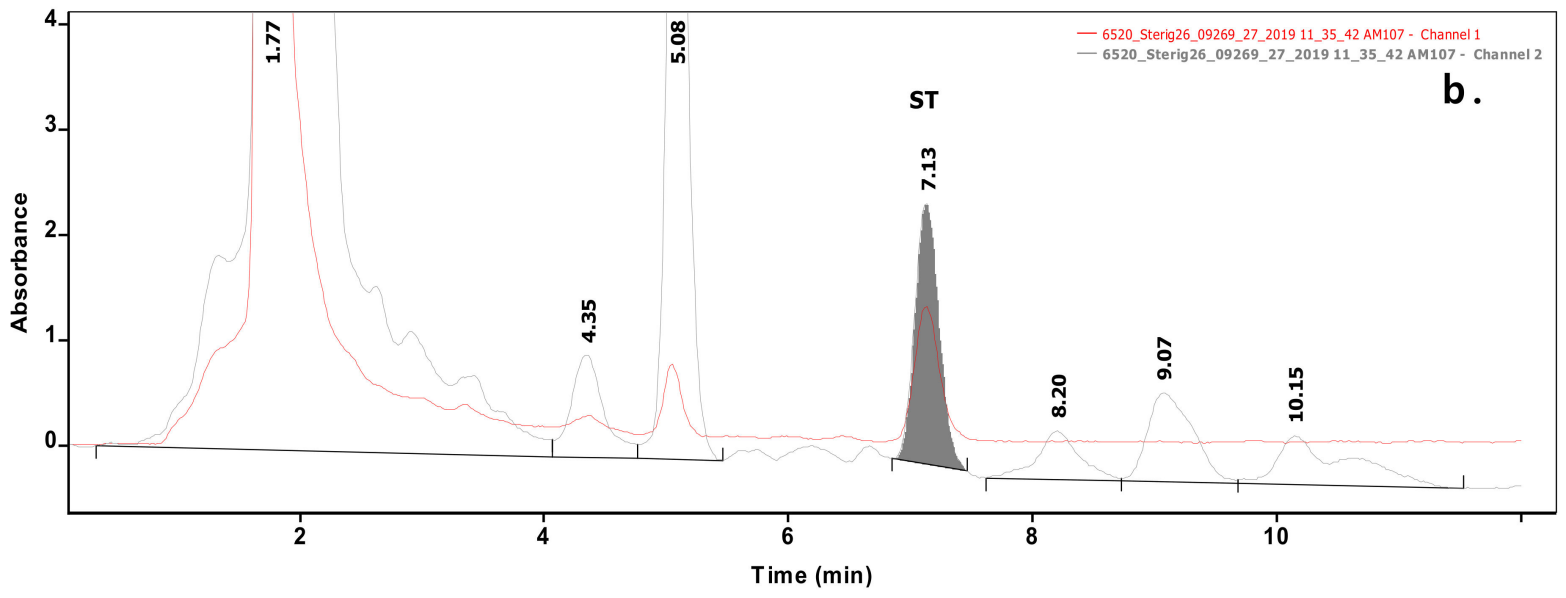
Chromatograms of samples extracted from biomass containing Aflatoxin B1 (AFB1) (**a**) or sterigmatocystin (ST) (**b**) at levels of 0.070 and 0.236 µg/mL, respectively.

**Figure 2:**
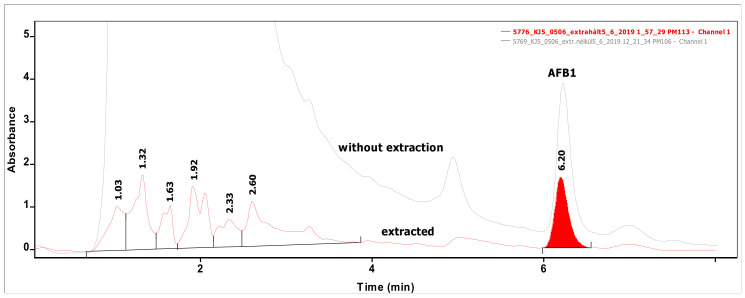
Chromatograms of a sample measured directly without any extraction (upper line) and that of the same sample extracted from MRS broth (lower line) containing AFB1 at the level of 0.50 µg/mL.

Analysis of 54 biomasses as well as the corresponding 6 MRS broth samples was carried out by high-performance liquid chromatography (HPLC) coupled with UV detection upon solvent extraction on the basis of a method optimization. Recent methods use mainly acetonitrile for the extraction of mycotoxins from foodstuffs, followed by cleanup with different modes of solid phase extraction (e.g., immunoaffinity, dispersive, etc.) to eliminate matrix effects. For cultivated bacteria or fungal strains, methanol [33] or chloroform [34,35] are the most frequently applied solvents, then the extracts are either subjected to a cleanup [33] or only filtered [35] prior to the analysis by a liquid chromatographic method. For complex matrices, gradient elution is applied [33], but for the bacteria investigated in the present study, a simple isocratic method gave sufficient separation. Typical chromatograms of target compounds are shown in Figure 1. The retention times were 6.07 and 7.13 min for AFB1 and ST, respectively. Although mycotoxins could be determined directly from the MRS broth, removal of the most polar matrix components resulted in better baseline and less interference (see Figure 2). For the extraction of MRS broth, the more volatile solvent, dichloromethane, allowed good recoveries (93.4 ± 3.1 and 97.6 ± 4.8% for AFB1 and ST, respectively), while for biomass, addition of 10% methanol to dichloromethane significantly increased the extraction efficiency by enhancing the solvent penetration to the cells. Ultrasound agitation seemed to be less effective than shaking of samples. Both analytes were determined from HPLC peak areas at the corresponding retention times with excellent linear calibration characteristics. For quantification of target compounds, peak areas determined for AFB1 at 365 nm and for ST at 240 nm were used. Peak purity for ST was checked by the ratios of signal intensities (peak areas) recorded at 240 and 325 nm, which was found 2.03 for standard solutions. The linear regression values of external calibration curves were 0.9992 and 0.9997, and the slopes were 110.7 and 145.3 for AFB1 and ST, respectively. The limits of detection, determined with standard solutions, were 0.010 µg/mL for both mycotoxins, and they were the same in spiked liquid matrices extracted from blank. 

### 2.2. Aflatoxin B1 Binding Capacities of LAB Other Then Lactobacilli

#### 2.2.1. Aflatoxin B1 Binding Capacities of *Enterococcus* Strains

**Figure 3:**
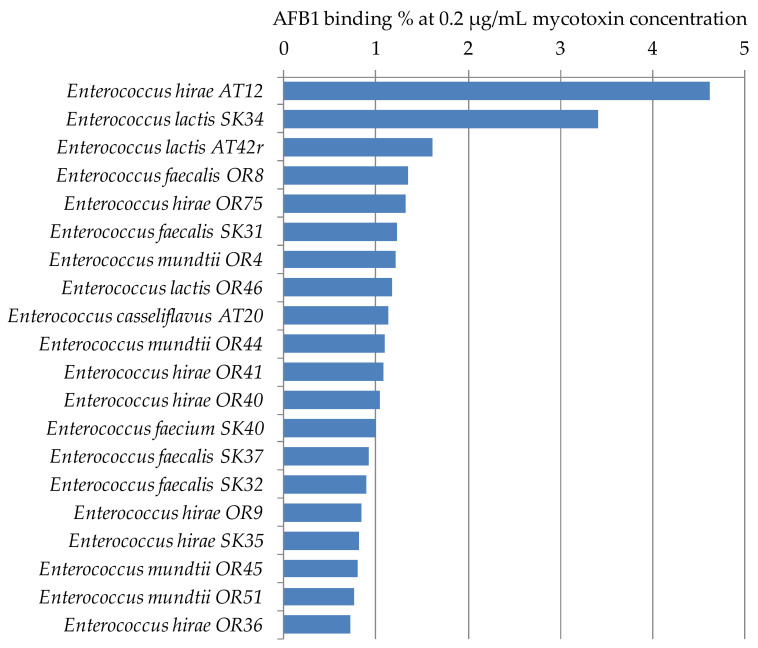
AFB1 binding capacities of *Enterococcus* strains at 0.2 µg/mL mycotoxin concentration in MRS broth.

Twenty *Enterococcus* strains from our lactic acid bacterium culture collection were selected for this study. One strain belonged to *E. casseliflavus*, 4 to *E. faecalis*, 1 to *E. faecium*, 6 to *E. hirae*, 3 to *E. lactis*, and 4 to *E. mundtii*. Two strains had higher AFB1 binding ability, *E. hirae* AT12 and *E. lactis* SK34 with 4.62% and 3.40%, respectively, for the other strains, the binding was below 1.61% (Figure 3). Regarding species, the best average AFB1 binding capacities were also obtained for species *E. lactis* and *E. hirae*, though for these two species, the standard deviations were higher than for the other species studied (Table 1). Juri et al. [36] found much higher AFB1 binding percentages for *Enterococcus faecium* GJ40 with 24–27% and 17–24%, and *E. faecium* MF4 with 36–42% and 27–32% at 0.05 and 0.10 µg/mL, respectively. The stability of those bacteria-AFB1 complexes formed was found to be high, up to 50% of AFB1 remained bound in bacterial cell after three washes with phosphate buffered saline. These differences in the results might be explained by the different strains or cultivation parameters and methods used in the studies; for example, in most studies, the bound mycotoxin concentration is calculated from the mycotoxin content remaining in the supernatant of the culture suspension, while in our investigations, the mycotoxin content of the biomass was determined directly.

#### 2.2.2. Aflatoxin B1 Binding Capacities of *Pediococcus* Strains

**Figure 4:**
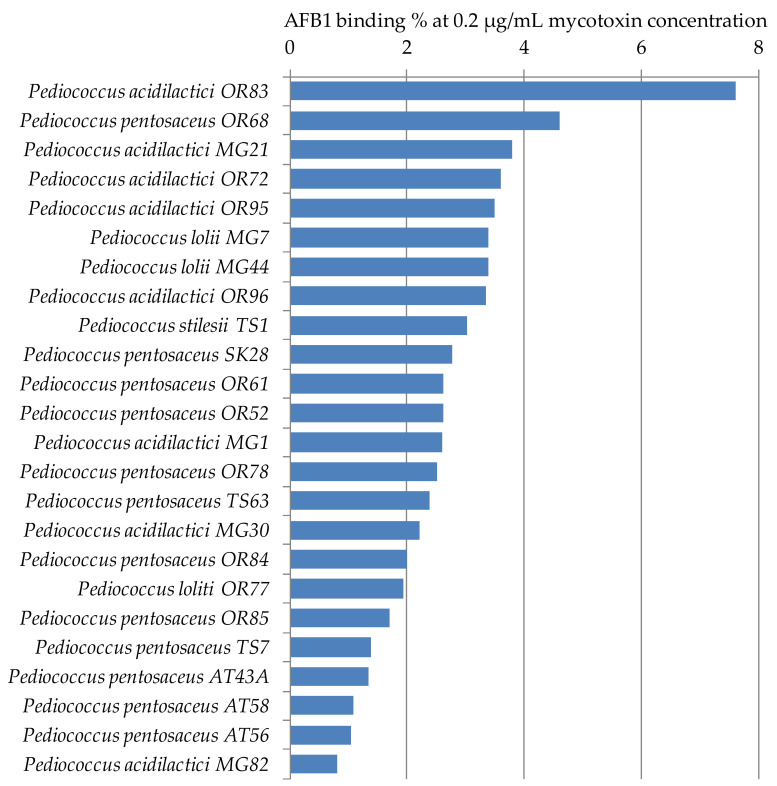
*Pediococcus* strains with percentage AFB1 binding capacities at 0.2 µg/mL mycotoxin concentration in MRS broth.

From the genus *Pediococcus*, the AFB1 binding capacities of 24 strains were studied. The strains belonged to species *P. acidilactici* (8 strains), *P. lolii* (3 strains), *P. pentosaceus* (12 strains), and *P. stilesii* (1 strain). According to the results shown in Figure 4, the best AFB1 binding ability was found for strain *P. acidilactici* OR83. For the other strains, the AFB1 binding percentages were around or below 4% (Table 2). The average binding capacities of the species were around 3% with the exception of *P. pentosaceus* at 2%. The highest standard deviation of the AFB1 binding abilities of the strains belonging to one species was obtained for *P. acidilactici*. These results are in agreement with data presented in the literature, where Zinedine et al. [37] found that *Pediococcus acidilactici* strain P55 removed 1.80% AFB1.

#### 2.2.3. Aflatoxin B1 Binding Capacities of *Lactococcus* and *Weissella* Strains

**Figure 5:**
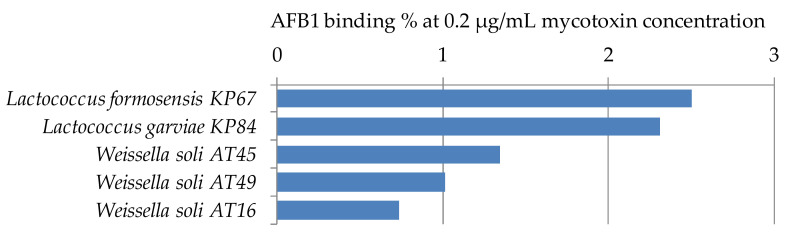
*Lactococcus* and *Weissella* strains with percentage AFB1 binding capacities at 0.2 µg/mL mycotoxin concentration in MRS broth.

For the study of AFB1 binding capacities of the genera *Lactococcus* and *Weissella*, only a limited number of strains has been used, one strain of *Lactococcus formosensis*, 1 strain of *L. garviae*, and 3 strains of *Weissella soli*. All studied strains have low mycotoxin binding capacities at the parameter setup of the experiment (Figure 5). Peltonen et al. [29] found that the three *Lactococcus lactis* strains studied bound 5.6 to 41.1% AFB1, which shows the wide range of binding capacities depending on the strains of a species. For aflatoxin binding of *Weissella* spp., only a few papers can be found in the literature. Binding with AFB1 was found to be 43.7% for *Weissella cibaria* NN20 by Nduti et al. [38] in skim milk at 10 ng/mL AFB1 concentration, also the EPS produced by *Weissella confusa* was proved to have aflatoxin binding capacity up to 34.79% at 100 mg/mL concentration of EPS, though no binding could be observed under 20 mg/mL EPS concentration [39]. Differences between our findings and the results might be explained by the different strains or methods used in the studies.

#### 2.2.4. AFB1 Binding Capacities of Lactic Acid Bacteria, Regarding Genus

Among the major genera belonging to lactic acid bacteria are *Enterococcus*, *Lactobacillus*, *Lactococcus*, *Pediococcus*, and *Weissella*. To compare the AFB1 binding capacities of the genera, averages, standard deviations, minimum and maximum values were calculated from the data obtained for the strains belonging to the same genus. Data obtained from our previous studies [32] were used to calculate the values for genus *Lactobacillus*. Results are presented in Table 3. The genus with the best AFB1 binding ability was the genus *Lactobacillus*, with an average binding of 3.16%. In addition, the standard deviation of the data for the abilities of its strains was the highest among genera, presenting a 20-fold difference between minimum and maximum values. The second best genus was genus *Pediococcus*, with average binding percentage of 2.72%, and the third place was taken by the genus *Lactococcus*. It can be concluded that for AFB1 bio-detoxification purposes, lactobacilli, pediococci, or lactococci should be chosen, but it is important to select a strain with better binding properties than the average value of their genera.

### 2.3. Sterigmatocystin Binding Capacities of Pediococcus Strains

**Figure 6:**
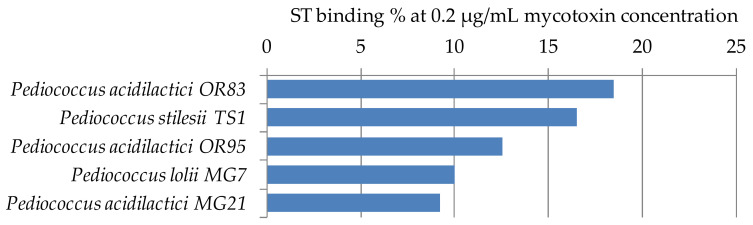
Sterigmatocystin binding capacities (%) of *Pediococcus* strains at 0.2 µg/mL mycotoxin concentration in MRS broth.

ST binding abilities of 5 *Pediococcus* strains of our LAB collection were determined according to Section 4.3. The results are summarized in Figure 6. Mycotoxin binding values were between 9–18%. These results are in agreement with our previous findings for *Lactobacillus* strains, that ST binding is 2–3 times the AFB1 binding capacity [32]. So far, no other results have been published in the literature that addressed the ST binding ability of lactobacilli.

## 3. Conclusions

For the analytical determination of target components, a simple isocratic separation was suitable after the appropriate sample preparation. Extraction of MRS broth by dichloromethane removed the polar matrix components, resulting in lower baseline and lower detection limit. Extraction of biomass required addition of 10% of methanol to dichloromethane to facilitate better release of target components from the surface of the cells. Accurate determination of mycotoxins in biomass is especially important in those cases when the binding capacity is low (e.g., 1%) and RSD values for the remaining mycotoxin in the MRS broth are comparable to those bound by the cells.

For the study of their AFB1 binding abilities, 49 lactic acid bacteria other than lactobacilli were selected from our culture collection. The results of our mycotoxin binding assays in MRS medium cannot be compared directly with PBS-based binding assays. However, it is a perfectly suitable method for determining the binding potential among our strains.

From the 20 *Enterococcus* strains belonging to 6 species, two had higher AFB1 binding ability, *E. hirae* AT12 and *E. lactis* SK34 with 4.62% and 3.40%, respectively. From the genus *Pediococcus*, the AFB1 binding capacities of 24 strains belonging to 4 species were studied, strain *P. acidilactici* OR83 stood out with a value of 7.60%, for the other pediococci, binding values of around 3% were obtained. For the genera *Lactococcus* and *Weissella*, low AFB1 binding capacities were found, though there was only limited number of strains studied. It can be concluded that for AFB1 bio-detoxification purposes, beside lactobacilli, pediococci can also be chosen, but it is important to select a strain with better binding properties than the average value of its genus. On the ST binding ability of strains belonging to the genus *Pediococcus*, the results are in agreement with our previous findings for *Lactobacillus* strains, that ST binding is 2–3 times the AFB1 binding capacity.

It should be noted that the best aflatoxin binding *Pediococcus* strain was the best ST binding, as well. This can be explained by the fact that the two structurally similar mycotoxins bind to the same cell wall polysaccharide (WPS) receptor of the bacterium. The binding strength may be stronger for ST than for AFB1. The different mycotoxin binding ability of strains of the same species, which can also be seen in the literature, may be due to their highly variable WPS cell wall components [40] rather than to the much more conserved peptidoglycan cell wall.

In this work, we report strong ST binding of non-lactobacillus LAB strains for the first time in the literature. The detection of different AFB1 and ST binding of LAB strains belonging to the same species with different binding activity may represent a model system that will allow the exploration of the exact molecular mechanism of the binding of these mycotoxins in the future.

## 4. Materials and Methods

### 4.1. Bacterial Strains

Forty-nine lactic acid bacterium strains of our collection isolated from feces samples of exotic herbivorous zoo animals were used for the studies (Table 4). The strains were identified by the 16S rDNA sequence extracted from pure bacterial cultures and sequenced by BaseClear (Leiden, The Netherlands). The LAB strains stored at −80 °C in 43.5% glycerin were thawed on ice before culturing.

### 4.2. Mycotoxins

AFB1 and ST were purchased from Sigma-Aldrich (Budapest, Hungary). Standard solutions were made by diluting the mycotoxin powder with methanol (puriss., MOLAR Chemicals Ltd., Halásztelek, Hungary) to make stock solutions of 50 µg/mL. The complete dissolution of the mycotoxins was ensured by mild heating and sonication (Ultrasonic Cleaning Instrument, Falc Instruments, Treviglio, Italy). The concentrations of the stock solutions were verified by HPLC measurement. These stock solutions were used in all experiments. The mycotoxin concentrations for our experiments were set at 0.2 µg/mL, which is the tenfold value of the maximum permitted level for AFB1 by EU Regulation No 574/2011.

### 4.3. Screening LAB Strains for Mycotoxin Binding Capacities

LAB strains were taken from −20 °C storage, thawed on ice, and 20 µl of the suspension was transferred to 9 mL lactic acid bacterium selective MRS (de Man, Rogosa and Sharpe, VWR) broth. The tubes were incubated at 37 °C for 24 h. Falcon tubes containing 15 mL of MRS broth were inoculated with 50 µl of the cultures. The tubes were incubated at 37 °C for two days. Three replicates were prepared with each strain.

After the incubation, the cell concentrations of the cultures were set at 10^8^ cfu/mL, and then 0.2 µg/mL of AFB1 or ST was added to the tubes. Pure MRS broth was used as negative control, and mycotoxin-only MRS broth without bacteria was used as positive control. The tubes were mixed by shaking and the tubes were incubated with the mycotoxin for 10 min at room temperature. The tubes were centrifuged at 4000 rpm for 40 min to separate the biomass from the supernatant. The supernatant was discarded [20]. The AFB1 and ST contents of the biomasses were determined by HPLC method described in Section 4.4.

### 4.4. Mycotoxin Extraction and HPLC Measurements

The amount of mycotoxin was determined by high performance liquid chromatography (HPLC) analysis using a YL9100 HPLC system equipped with a YL9150 autosampler (YL Instruments, Gyeonggi, Korea). For the measurement, the mycotoxin was extracted from the samples by the following steps. For the extraction of the mycotoxin from the biomass, 1.8 mL of dichloromethane and 0.2 mL of methanol were added to the Falcon tube containing the biomass, using the ratio that gave best results in preliminary experiments. The mixture was pipetted into Eppendorf tubes. The tubes were vortexed in a horizontal shaker for 20 min in the dark and then centrifuged at 3000 rpm for 10 min. One ml of the supernatant was taken out and the solvent was evaporated to the dryness in a clean Eppendorf tube at 45 °C under a fume hood in a Thermo Shaker (TS-100, Biosan, Riga, Latvia). MRS broth was extracted similarly, but 1 mL of dichloromethane was shaken with one milliliter of supernatant for 20 min. From the dichloromethane phase, 0.5 mL was taken out and concentrated in a clean Eppendorf tube at 45 °C under a fume hood. The residues were solved in 1 mL of eluent (see below) and the sample was filtered through a 0.45 μm hydrophilic polytetrafluoroethylene (PTFE) syringe filter (Labex Ltd., Budapest, Hungary) prior to HPLC determination.

The mycotoxin content of the samples was determined by UV detection (HPLC-UV) after an isocratic liquid chromatographic separation. UV detector signals were recorded at λ = 365 nm or λ = 240 and 325 nm for AFB1 and ST, respectively. The separation was performed on a Brisa (Technochroma, Barcelona, Spain) C18 column (5 µm, 15 cm × 0.46 cm) at 30 °C. The eluent flow rate was set to 1.0 mL/min and 30 µl of samples were injected. The eluent consisted of 60:20:20 = A:B:C eluents, and 40:30:30 = A:B:C eluents (A = 90% water: 10% MeOH, B = MeOH, C = Acetonitrile), held till 8 and 12 min for AFB1 and ST, respectively. Extracts of blank non-spiked control biomass did not contain interfering matrix components, therefore quantitation was based on instrumental (external) calibration with standard solutions in the range between 0.010 and 2.00 µg/mL. Recoveries at concentration of 0.2 µg/mL in the spiked samples were determined by adding a known concentration of AFB1 or ST to the liquid of blank samples. Peak purities were systematically checked by recording absorption at two wavelengths, and peak area ratios at those wavelengths were compared to the ratios characteristic to standard solutions of the analyte (ST). Binding capacities (%) were calculated on the basis of analyte concentrations in the extracted biomass samples related to the initial MRS broth levels considering the corresponding concentration factor applied (see sample preparation, above). RSD values calculated from the three parallel injections of standard solutions ranged between 0.2 and 1.4%.

## Figures and Tables

**Table 1:** Percentage AFB1 binding capacities of *Enterococcus* species at 0.2 µg/mL mycotoxin concentration in MRS broth.

Species	Number of Strains	Average Binding %	STD	Min Binding %	Max Binding %
*Enterococcus lactis*	3	2.06	1.18	1.17	3.40
*Enterococcus hirae*	7	1.49	1.39	0.72	4.62
*Enterococcus casseliflavus*	1	1.14		1.14	1.14
*Enterococcus faecalis*	4	1.10	0.23	0.89	1.35
*Enterococcus faecium*	1	1.00		1.00	1.00
*Enterococcus mundtii*	4	0.97	0.22	0.77	1.21

**Table 2:** Percentage AFB1 binding capacities of *Pediococcus* species at 0.2 µg/mL mycotoxin concentration in MRS broth.

Species	Number of Strains	Average Binding %	STD	Min Binding %	Max Binding %
*Pediococcus acidilactici*	8	3.43	1.95	0.80	7.60
*Pediococcus stilesii*	1	3.03		3.03	3.03
*Pediococcus lolii*	3	2.90	0.84	1.93	3.39
*Pediococcus pentosaceus*	12	2.18	0.99	1.05	4.60

**Table 3:** Percentage AFB1 binding capacities of lactic acid bacteria, regarding genus, at 0.2 µg/mL mycotoxin concentration in MRS broth (*Lactobacillus* results are from previous studies [32]).

Genus	Number of Strains	Average Binding %	STD	Min Binding %	Max Binding %
*Lactobacillus*	105	3.16	1.98	0.55	11.50
*Pediococcus*	24	2.72	1.42	0.80	7.60
*Lactococcus*	2	2.40	0.14	2.31	2.50
*Enterococcus*	20	1.35	0.96	0.72	4.62
*Weissella*	3	1.03	0.31	0.73	1.35

**Table 4:** Strains of lactic acid bacterium species of our collection used in the current study.

Species	Strains
*Enterococcus*	
*E. casseliflavus*	AT20
*E. faecalis*	OR8, SK31, SK32, SK37
*E. faecium*	SK40
*E. hirae*	AT12, OR9, OR36, OR40, OR41, OR75, SK35
*E. lactis*	AT42r, OR46, SK34
*E. mundtii*	OR4, OR44, OR45, OR51
*Lactococcus*	
*L. formosensis*	KP67
*L. garviae*	KP84
*Pediococcus*	
*P. acidilactici*	MG1, MG21, MG31, MG82, OR72, OR83, OR95, OR96
*P. lolii*	MG7, MG44, OR77
*P. pentosaceus*	AT43A, AT56, AT58, OR52, OR61, OR68, OR78, OR84, OR85, SK28, TS7, TS63
*P. stilesii*	TS1
*Weissella*	
*W. soli*	AT16, AT45, AT49
